# Surgery for rheumatic mitral valve disease in sub-saharan African countries: why valve repair is still the best surgical option

**DOI:** 10.11604/pamj.2016.24.307.7504

**Published:** 2016-08-11

**Authors:** Charles Mve Mvondo, Marta Pugliese, Alessandro Giamberti, David Chelo, Liliane Mfeukeu Kuate, Jerome Boombhi, Ellen Marie Dailor

**Affiliations:** 1 Division of Cardiac surgery, Cardiac Centre of Shisong, Kumbo, Cameroon; 2 Divison of Cardiac Surgery, University of Rome Tor Vergata, Rome, Italy; 3 Department of Pediatrics, Chantal Biya Foundation, Yaoundé, Cameroon; 4 Department of Internal Medicine, Yaoundé Central Hospital, Yaoundé, Cameroon; 5 Department of Cardiology, Yaoundé General Hospital, Yaoundé Cameroon; 6 Department of Anesthesiology, University of Rochester School of Medicine and Dentistry, Rochester, New York, USA

**Keywords:** Rheumatic heart disease, mitral valve repair, valve replacement

## Abstract

Rheumatic valve disease, a consequence of acute rheumatic fever, remains endemic in developing countries in the sub-Saharan region where it is the leading cause of heart failure and cardiovascular death, involving predominantly a young population. The involvement of the mitral valve is pathognomonic and mitral surgery has become the lone therapeutic option for the majority of these patients. However, controversies exist on the choice between valve repair or prosthetic valve replacement. Although the advantages of mitral valve repair over prosthetic valve replacement in degenerative mitral disease are well established, this has not been the case for rheumatic lesions, where the use of prosthetic valves, specifically mechanical devices, even in poorly compliant populations remains very common. These patients deserve more accurate evaluation in the choice of the surgical strategy which strongly impacts the post-operative outcomes. This report discusses the factors supporting mitral repair surgery in rheumatic disease, according to the patients' characteristics and the effectiveness of the current repair techniques compared to prosthetic valve replacement in developing countries.

## Introduction

Although mitral valve repair (MVR) has become the treatment of choice in degenerative etiology, the fundamentals of this surgery promoted by Carpentier [1] were developed in the rheumatic heart disease (RHD) era. In fact, "The French Correction" paper reported rheumatic mitral pathology in about sixty percent of the patients, and nearly ninety percent of children with rheumatic mitral lesions were eligible for valve repair. These reconstructive techniques were desirable in patients coming from low-income regions, where post-surgical management of prosthetic valves could have been suboptimal. Thirty years after Carpentier's paper, RHD is still endemic in many poor regions such as sub-Saharan Africa; however, prosthetic mitral replacement (PVR) especially with mechanical devices has become the preferred option because repair of rheumatic valves is often associated with a high rate of failure and reoperation [2–4]. Furthermore, since acute rheumatic fever (ARF) and RHD are becoming rare in western regions, scientific interest and surgeon experience with this aetiology are lacking [5], with numerous patients undergoing PVR, despite possible eligibility for valve repair. Given the known advantages of MVR over PVR [6–8], and the evolving techniques of mitral valve repair which have recently reported encouraging results in rheumatic lesions [9–11], the widespread use of prosthetic valves in the complex socio-cultural scenario of sub-Saharan regions is becoming more questionable. Therefore, it is appropriate to revisit the recommended surgical treatment, according to the patients' characteristics and the effectiveness of the current repair techniques as compared to PVR in these developing countries.

## Methods

A literature search was conducted in medical databases Pubmed and Google Scholar using the following combination of keywords: "rheumatic heart disease", "mitral valve repair", "mitral valve replacement", "developing countries", "anticoagulation". There were no restrictions to language, date of publication or publication status. All identified citations were critically evaluated to establish the possible relevance of the articles for inclusion in the review. A total of 46 papers were finally analyzed. The bibliographies of the articles in hand were used to find other references. Attention was given to articles comparing the outcomes of mitral valve repair and replacement for rheumatic heart disease.

## Current status of knowledge

### Predominance of RHD in young and female populations

According to estimates of the World Health Organization, about 16 million people are affected by RHD worldwide, with a high prevalence among young populations in developing countries [12]. The sub-Saharan African regions have one of the highest prevalence of RHD which is estimated about 30/1000 children [13], and with a great number of affected patients being women. Indeed, a high prevalence of rheumatic mitral disease in female sex has been reported in various studies [14–15] and a high incidence of rheumatic valve disease has been found in pregnant women living in low-income countries [16–18], demonstrating the poor awareness of these populations of the risks represented by cardiovascular diseases, including those associated with prosthetic heart valves. Many of these patients are of child-bearingage with a fertility rate estimated between 4 and 7 children per woman in Sub-Saharan Africa which is the highest in the world. This cultural reality, in addition to the high rates of illiteracy and the lack of contraceptive strategies, means that many will probably face pregnancy after PVR despite the contrary recommendations of their physicians, increasing the risk of fetal and maternal complications associated in part to lifetime anticoagulation therapy. Thus, candidates for mitral valve surgery in the Sub-Saharan region are often patients with high life expectancy and fertility rate that requires a careful evaluation in the choice of the surgical strategy.

### Patient's survival after mitral surgery: MVR versus PVR

It is widely ascertained that PVR reduces life expectancy, especially in children and young adults regardless of native valve pathology, due to prosthetic valve-related complications [19–22]. A recent study by Brown et al. [19] reported a 35 year actuarial survival of 71% after mitral PVR in pediatric patients, which is quite low if considering the young age of the study cohort (mean age: 8 years ; range: 2 weeks to 18 years). These results were consistent with those of De Santo et al. [20] who reported a 25 year survival of 70% in young women (mean age: 30 years) after mechanical mitral replacement. Other authors have described similar data, reporting a mean survival in the range of 63 to 66% over 10 to 15 years of follow up [21, 22]. Furthermore, the rate of reoperation has been found to be high in young patients after PVR due mainly to prosthetic valve mismatch caused by somatic growth and valve thrombosis [19–23]. Brown et al. [19] reported a 37% reoperation rate during a 35-year follow-up period, caused mainly by prosthesis mismatch, due to the use of small prosthesis size during the first operation. Similar results were observed by Alsoufi and colleagues [24] reporting a 20-year survival and freedom from reoperation of only 74% and 49% respectively with half of patients were re-operated within 20 years of their first surgery (mean age at first surgery: 11.4 years). Conversely, valve repair is associated with improved survival when compared to PVR [25–27], and despite the high rate of reoperation associated with rheumatic MVR, reconstructive techniques might increase survival in these young patients. Yau and colleagues [7] found that when compared to PVR, MVR was a predictor of better survival even in case of reoperation, and these advantages were independent of age or other preoperative characteristics. They conclude that rheumatic mitral valves should be repaired when technically feasible, accepting a risk of reoperation, to maximize survival and reduce morbidity. Similar conclusions by Brown et al. advocated the use of conservative approaches in young patients with mitral disease, reserving PVR for medical and repair failure because reoperation, prosthetic valve related complications and late mortality remain high in young patients [**19**].

### Pathophysiologic patterns of rheumatic mitral disease in developing countries are favourable to valve repair

Clinical and surgical patterns of rheumatic mitral valve disease may differ between patients from western and southern regions. In the developed countries, patients are often older, some with previous surgical or percutaneous procedures for mitral disease especially for valve stenosis [28]. These patients generally have severe fibrotic and calcified valves, which are not amenable to valve repair. Their advanced age, the high rate of chronic atrial fibrillation and the good post-surgical management in the developed world makes PVR a reasonable option in this subgroup. Young patients are the most affected by RHD in the developing regions with pure mitral regurgitation and mixed lesions being the most common valve dysfunctions. Nkomo et al. [29] confirmed this hypothesis, reporting a prevalence of pure MR during the first and second decades of life in patients with rheumatic mitral disease. Similar data were previously published by Marcus et al. [30] who described the demographic, pathologic, and hemodynamic profiles of patients with severe rheumatic mitral valve disease in a developing country, and found that pure regurgitation was the most common lesion in the first and second decades whereas the relative prevalence of pure stenosis and severe tissue lesions increased with age. Therefore, the grade of fibrosis is often limited in young patients at the time of diagnosis, allowing reconstructive techniques. Duran et al. [3] found that the repair rate in rheumatic mitral disease was related to the patient's age (76.6 % < 20 years, 59.1% > 20 < 40, 33.8% > 40 years). Similarly, Chauvaud et al. [31] described a more advanced mitral disease (described as type IIIa of Carpentier functional classification) in older patients whereas type II was predominant in the youngest age, with strong correlation with repair results.

### Improvement of repair techniques

The high rate of failure and reoperation associated with MVR in rheumatic aetiology has been commonly argued to favour PVR in this disease. This thinking developed during the early era of mitral reconstruction techniques, as various studies had reported suboptimal repair results [32, 33]. Therefore, many surgeons have been trained with the idea that rheumatic MVR was not advisable. On the other hand, as the experience in MVR increased, RHD significantly decreased in western regions where the main aspects of cardiac surgery were developed, with few institutions developing extensive experience in rheumatic valve surgery. Moreover, the characteristics of rheumatic valve disease in the developed world and the good results of new generation prosthetic valves have definitively extended the preference of PVR in such patients. Unfortunately, this approach has been "transferred" in many developing countries, where PVR has become the most common treatment even in cases suitable to repair. Indeed, in a 15-year experience with mechanical valve replacement by a West African group, 96% of the patients who underwent mechanical PVR had pure MR at the time of surgery [34]. With few exceptions, this report reflects the current approach in many sub-Saharan centers, despite the scarcity of published clinical data. However, more has been learned from emergent countries such as India representing "hybrid" social environment, characterized by high rate of RHD as observed in other developing countries but good standards in cardiac surgery. Clinical studies from these areas have further contributed to understanding how evolving concepts and current repair techniques could improve long-term results in rheumatic mitral repair [35, 36]. In fact, techniques of MVR have evolved during the last 3 decades, improving results in the field of rheumatic lesions as reported by several studies [9, 10, 31, 37, 38]. A good understanding of standardized techniques in the early 2000's have gradually led to better results as compared to the early 1980's. For instance, prosthetic ring annuloplasty has become a mainstay of MVR, despite the initial reluctance and confused data reported in the early era of mitral reconstruction. Nunley and colleagues [39], reporting their experience on MVR between the 60's and 80's, suggested that ring annuloplasty was not effective and was potentially causing harm. Conversely, long-term studies have successively demonstrated the importance of this technique in MVR, and more is now known about the effectiveness of the different types of annuloplasty rings (pericardial strip, flexible, rigid, complete or semi-complete). Moreover, the so-called non-classical techniques (leaflet augmentation with pericardial patch, mitral homograft, neo-chordae implantation, or leaflet reconstruction with tricuspid autograft) have further extended the indications for MVR, especially in rheumatic etiology with many experienced centers reporting good long-term results. Chauvaud et al. [31] reported their 29-year results in rheumatic MVR, with a 20-year actuarial survival and freedom from reoperation of 82±18% and 55±25% respectively. Another study by El Oumeiri et al [10] reports 8-year freedom from cardiac death and reoperation of 98±2% and 94±5% respectively. The authors used an aggressive approach including non-classical techniques, reporting 83±9% freedom from significant mitral regurgitation at 8 years. These results seem to be better than previous reports where freedom from reoperation was less than 75% at 5 years [32, 33]. This new trend consists of the full resection of all fibrotic tissue followed by partial or complete valve reconstruction with autologous or heterologous material. According to many authors, this strategy may increase the repair rate in addition to ensure better durability by the removal of fibrotic tissue, as residual fibrotic tissue strongly correlates with disease progression leading to repair failure over time.

### Advances in perioperative patient care

Perioperative management of patients' undergoing valve surgery has also improved, with more accurate pre-operative assessment of valve function, routine use of intraoperative transoesophageal echocardiography, and better myocardial protection strategies. The surgeons can be less concerned with cross-clamping duration and may first attempt MVR and then PVR only in case of suboptimal repair. Furthermore, many experts have shared their methods to assess repair feasibility and to prevent failure. Gupta and colleagues [36] described an interesting method to predict repair feasibility by measuring the length of the anterior mitral leaflet (AML), predicting valve reparability with 97% sensitivity and 100% specificity. According to the authors this data could orient the surgical strategy with the addition of specific techniques such as leaflet patch extension in the case of small anterior mitral leaflet length (<18mm/ m2). One more empirical method is the "rebound-like effect" of the AML, assessed by pulling this leaflet in the left ventricle; its rebound demonstrates a severe grade of leaflet fibrosis that contraindicates repair [40]. Despite the heterogeneity of these reports and results, they add useful information to the armamentarium of the modern cardiac surgeon dealing with rheumatic valves, allowing a more confident approach as compared to some decades ago.

### Post-surgical follow-up of patients with prosthetic valves in developing countries is challenging

More than fifty years ago, Frater said, "A patient with a mitral prosthesis is a patient for life," [41] emphasizing the permanent risk of cardiac events and mortality due to prosthetic valve-related complications. These have been estimated to be about 2%/patient/year [42], and their incidence may be strongly influenced by the patient socio-cultural environment [43, 44]. Advances in medical care, financial resources, and better education of patients in western countries have yielded optimal compliance with therapy with acceptable long-term results, whereas this is not yet the case in the low-income regions. The necessity of avoiding anticoagulant therapy in poorly compliant populations has been an important factor to recommend conservative approaches since the earliest period of reconstructive valve surgery. This remains an important consideration in many African developing countries where the socio-cultural conditions have not really improved. Local health care systems are lacking as demonstrated by the high incidence of ARF and RHD in these regions which result from both the poor living conditions and the lack of effective preventive measures. It is then challenging to ensure adequate follow-up of patients with PVR in these conditions with limited health care assistance [45] and medical personnel [46] in addition to factors such as the high rate of illiteracy and infectious diseases. Potentially, a great number of patients will not adequately pursue the recommended follow-up or will simply discontinue the anticoagulant therapy because they are "feeling good", exposing themselves to fatal complications. The acute nature of these events will often lead to death due to the delay in diagnosis, and inadequate medical assistance.

## Conclusion

Introducing his paper [1] Carpentier said, "It's not enough to save patient lives, we must also take into consideration the quality of life given to the patient and the socio-economic impact of our surgical actions." This clearly emphasizes the importance of adapting the surgical treatment to the patient. Especially in complex socio-cultural realities and environment, this topic deserves more critical analysis and evaluation from medical specialists to avoid indiscriminate and erroneous application of any "standard" surgery.

### What is known about this topic

- Advantages of mitral valve repair over prosthetic valve replacement in degenerative etiology are well established;
- Mitral valve repair in rheumatic disease is associated with high rate of failure and reoperation as compared to degenerative disease;
- Management of patients with prosthetic valves is challenging in developing regions for the limited financial and medical facilities especially for anticoagulation control.

### What this study adds

- Rheumatic mitral lesions in young patients are often amenable to repair;
- Recent experiences of rheumatic mitral repair have reported improved freedom from failure and reoperation on the long term as compared to earliest studies;
- With few exceptions, the management of patients with prosthetic valves especially mechanical valves in the Sub-Saharan regions remains challenging.
